# Impact of Fermented Wheat Bran Dietary Fiber Addition on Dough Rheological Properties and Noodle Quality

**DOI:** 10.3389/fnut.2022.952525

**Published:** 2022-07-07

**Authors:** Ling Fan, Li Li, Anmin Xu, Jihong Huang, Sen Ma

**Affiliations:** ^1^Food and Pharmacy College, Xuchang University, Xuchang, China; ^2^College of Food Science and Engineering, Henan University of Technology, Zhengzhou, China; ^3^State Key Laboratory of Crop Stress Adaptation and Improvement, College of Agriculture, Henan University, Kaifeng, China

**Keywords:** wheat bran, dietary fiber, fermentation, protein network, noodle quality

## Abstract

This study aimed to evaluate the effect of fermented wheat bran dietary fiber (FWBDF) on the rheological properties of the dough and the quality of noodles and to compare it with the effect of the unfermented WBDF (UWBDF). WBDF was fermented with *Auricularia polytricha*. The results showed that adding UWBDF/FWBDF increased the storage modulus *G'* and loss modulus *G”* of the dough, converted α-helices and β-turns into β-sheets and random coils, respectively, inhibited water flow, increased cooking loss, and decreased the maximum resistance in the noodles. The formed gluten network had a more random and rigid structure, resulting in the deterioration of the quality of noodles. Furthermore, the number of α-helices and the peak proportions of weakly bound water *A*_22_ increased but the number of β-sheets and cooking loss decreased in the FWBDF group compared with the UWBDF group. FWBDF (≤4%) improved the hardness of noodles, while UWBDF decreased it. These changes indicated that fermentation could reduce the destructive effects of WBDF on the quality of noodles, providing a new perspective on balancing dietary fiber-rich and high-quality foods.

## Introduction

Developing food products with high dietary fiber content has become an effective way to change the dietary pattern of consumers for meeting the growing demand for healthy food. An increase in the consumption of dietary fiber is recommended in most European countries, as adequate intake of dietary fiber can improve human health and the management of health diseases (1, 2). Dietary fiber is an important natural carbohydrate polymer that is major component of plant cell walls and is not digested or absorbed by the small intestine. Due to its porous physical structure and the abundant hydroxyl groups in the side chain, dietary fiber has good water retention and hydrogen bond forming capabilities (3). Moreover, in the plant cell walls, phenolic compounds are always combined with the backbone of dietary fiber, which gives dietary fiber good antioxidant capacity (2, 4). In addition, dietary fiber can selectively stimulate the activity or growth of probiotic bacteria in the colon, thereby improving the microbial balance in the gut, which in turn has a beneficial effect on the health of the host (2, 5). Also, dietary fiber effectively prevents colonic diseases, loses weight, lowers cholesterol, and regulates gut microbe metabolism and intestinal microbial metabolism (6, 7).

Recently, the trend is to enhance the dietary fiber content of traditional flour-based products, such as noodles. However, dietary fiber is associated with the low quality of wheat flour products based on the interaction between dietary fiber and the gluten matrix. Many researchers focused on modifying the properties of dietary fiber to reduce its destructive effects on end-use products while anticipating the desirable effects on its physiological and functional properties (8–10). Several studies have emphasized that fermentation is an effective way to improve the properties of wheat bran, thus increasing the content and bioavailability of the functional compounds such as soluble fiber and phenolic substances, degrading antinutritive factors such as phytic acid, and significantly improving the antioxidant capacity (11–13). Meanwhile, fermentation can effectively reduce the side effects of bran on bread volume, positively affecting the overall characteristics of the final bread (14, 15). The fermentation of *Fomitopsis pinicola* can not only increase the contents of total phenol and alkylresorcinols of wheat bran but also improve the textural properties of the dough and bread (16).

*Auricularia polytricha* (*A. polytricha*), an edible fungus classified as a Basidiomycota, has been shown to efficiently degrade lignocellulose *via* its oxidative ligninolytic systems. At present, edible fungi are recognized as safe strains producing various enzymes such as cellulase and amylase during the fermentation process (17). Also, these enzymes can improve the chemical composition and biological activity of the substrates. *A. polytricha* is reported to have many functions such as anti-oxidation, tumor suppression, and anti-nociceptive activity; it has been widely used as a healthy food in Oriental countries, especially in China and Korea (18). Lignin creates the recalcitrant and complex structure in WBDF by filling the spaces around the cellulose and hemicellulose structures and binding them together, thus serving as a barrier to the formation of the gluten network structure (19). Furthermore, wheat bran dietary fiber (WBDF) can cause deterioration in the quality of products due to its specific structure. *A. polytricha* may be a good solution to the aforementioned issue. However, no reports have emphasized the effect of *A. polytricha*–fermented WBDF (FWBDF) on the rheological properties of dough and the quality of noodles.

In the present study, WBDF was fermented using the *A. polytricha* strain. The effects of FWBDF on the dynamic rheological properties of dough, structural properties of gluten, water distribution, cooking, texture, and extension properties of noodles were evaluated and compared with those of unfermented WBDF (UWBDF). This study provided new prospects for balancing dietary fiber-rich and quality foods with fermented dietary fiber, and facilitated the basic theory for the comprehensive utilization of fermented dietary fiber in the food industry.

## Materials and Methods

### Materials

Wheat flour was purchased from Zhengzhou Jinyuan Noodles Industry Co. (Henan, China). Wheat bran was purchased from Henan Zhonghe Agricultural Development Group Co., Ltd. (Henan, China). *A. polytricha* 5.584 (CGMCC 5.584), an edible jelly fungus, was supplied by China General Microbiological Culture Collection Center (CGMCC, Beijing, China). The activity of laccase production by *Auricularia polytricha* 5.584 was 188.54 U/mL, and the degradation rate of lignin from wheat bran reached 68.72% (20).

### Preparation of UWBDF and FWBDF

UWBDF and FWBDF were prepared by the method proposed by Jiang et al. (21) with some modifications. The powdered wheat bran was suspended in ultrapure water (1:10, *w*/*v*) for 30 min with gentle stirring at 95°C. The pH of the mixture was adjusted to 5.6 with HCl and then 1.5% (*w*/*w*) of thermostable α-amylase (40,000 U/g) was added to react for 30 min at 95°C. After the temperature was dropped to 50°C, the pH of the mixture was adjusted to 9.0 with NaOH and 3% (*w*/*w*) alkaline protease was added (≥2,00,000 U/g) to react for 2 h. The mixture was centrifuged at 4,390 g for 20 min. The supernatant was removed and filtered with ultrapure water until the filtrate was clarified. The resultant precipitate was immediately dried in an oven for 24 h at 60°C and then ground through a 150-μm mesh screen to obtain UWBDF. Ten milliliters of the *A. polytricha* 5.584 culture solution (188.54 U/mL) was inoculated into 100 mL of UWBDF-containing fermentation medium (41.5 g/L) and incubated at 26°C in a shaker for 15 days. The FWBDF was dried in an oven at 60°C and then ground through a 150-μm mesh screen.

### Preparation of Dough, Gluten, and Noodles

The noodles were made from 100 g of total flour and 38 g of distilled water (30°C). Zero percent (control), 2, 4, 6, 8, and 10% UWBDF and FWBDF were added based on the total weight of the flour. The flour was stirred using a JHMZ200 pin mixer (East Fude Technology Development Center, Beijing, China) for 7 min at 104 rpm. The dough was put into a plastic bag and allowed to rest for 30 min at 30°C. It was divided into three groups: the first was sheeted with a JMTD168/140 sheeting machine (Beijing Dongfu Jiuheng Instrument Technology Co., Ltd., Beijing, China) for the rheological measurement of dough; the second was washed with distilled water to obtain the gluten, freeze-dried, and ground through a 150-μm mesh sieve; and the third was sheeted and then cut into strips to obtain the noodles (thickness 1.25 mm, width 2.0 mm).

### Dynamic Rheological Properties

The dynamic rheological properties of dough were determined using a RheoStress 6,000 rotational rheometer (Thermo Fisher Haake, Hamburg, Germany) with a 20-mm diameter steel plate (2-mm gap) by the method proposed by Guadarrama et al. (22) with some modifications. A frequency sweep test was performed from 0.1 to 10 Hz at 25°C. A creep and recovery test was performed under the following conditions: fixed stress 100 Pa, the creep phase lasting 180 s, and the recovery phase lasting 300 s.

### Fourier-Transform Infrared Spectroscopy

The gluten structure was measured using a Fourier-transform infrared spectrometer (Nicolet iS50, Thermo Fisher Scientific, Waltham, MA, USA) as described by Zhan et al. (23). The freeze-dried gluten sample (2 mg) and predried potassium bromide (200 mg) were fully ground and pressed into thin slices. The spectra in the range of 4,000–400 cm^−1^ were recorded with 32 scans per spectrum at a resolution of 4 cm^−1^. The structure information of gluten was analyzed using PeakFit V4.12 software (SPSS Inc., Chicago, IL, USA).

The most sensitive spectral region for gluten was the amide I band (1,600–1,700 cm^−1^), where C–O was the stretching vibration of the peptide linkages. The β-sheets (1,610–1,640 cm^−1^), random coils (1,640–1,650 cm^−1^), α-helices (1,650–1,660 cm^−1^), and β-turns (1,660–1,670 cm^−1^) were the protein secondary structures with the corresponding ranges of the amide I (23, 24).

### Low-Field Nuclear Magnetic Resonance

The noodle sample (3 g) was cut and placed in a test tube. The water distribution of noodles was measured using a low-field nuclear magnetic resonance system (VTMR20-010V-T, Shanghai Niumai Electronic Technology Co., Ltd., Shanghai, China) by the experimental method proposed by Yu et al. (25) with some modifications. The number of sampling points (TD) was 66,666, the sampling frequency (SW) was 333.33 kHz, the sampling interval time (TW) was 3,000 ms, the number of echoes was 2,000, the echo time was 0.1 ms, and the number of repeated scans was 16.

### Cooking Property

The fresh noodles were boiled in 400 mL of boiling water until the optimum cooking time was reached, quickly removed, and rinsed with cold water for 30 s as described by Sandhu et al. (26) with some modifications. Then, the cooking water was collected in a volumetric flask and mixed with 500 mL of water. The aliquots of 100 mL were dried to constant weight at 105°C. The residue was weighed and reported as a cooking loss.

### Texture and Extension Properties

An A-XT2i texture analyzer with an HDP/PFS probe (Stable Micro Systems, London, England) was used to analyze the texture of cooked noodles by the method described by Gao et al. (27). The pretest, test, and post-test speeds were 2.0, 0.8, and 0.8 mm/s, respectively. The interval time of compressions was 1 s, the strain rate was 75%, and the trigger force was 10 g. The extensional test was carried out using an A/SPR probe, and the distance and trigger force were 100 mm and 0.5 g, respectively. The analyses were repeated six times for each sample.

### Statistical Analysis

The data were expressed as the mean ± standard deviation of triplicate replications. SPSS 16.0 was used to perform the analysis of variance and significant difference test (Duncan, *P* < 0.05), and Origin 8.5 was used for plotting.

## Results and Discussion

### Dynamic Rheological Properties of Dough

#### Frequency Sweep

**Figure 1:**
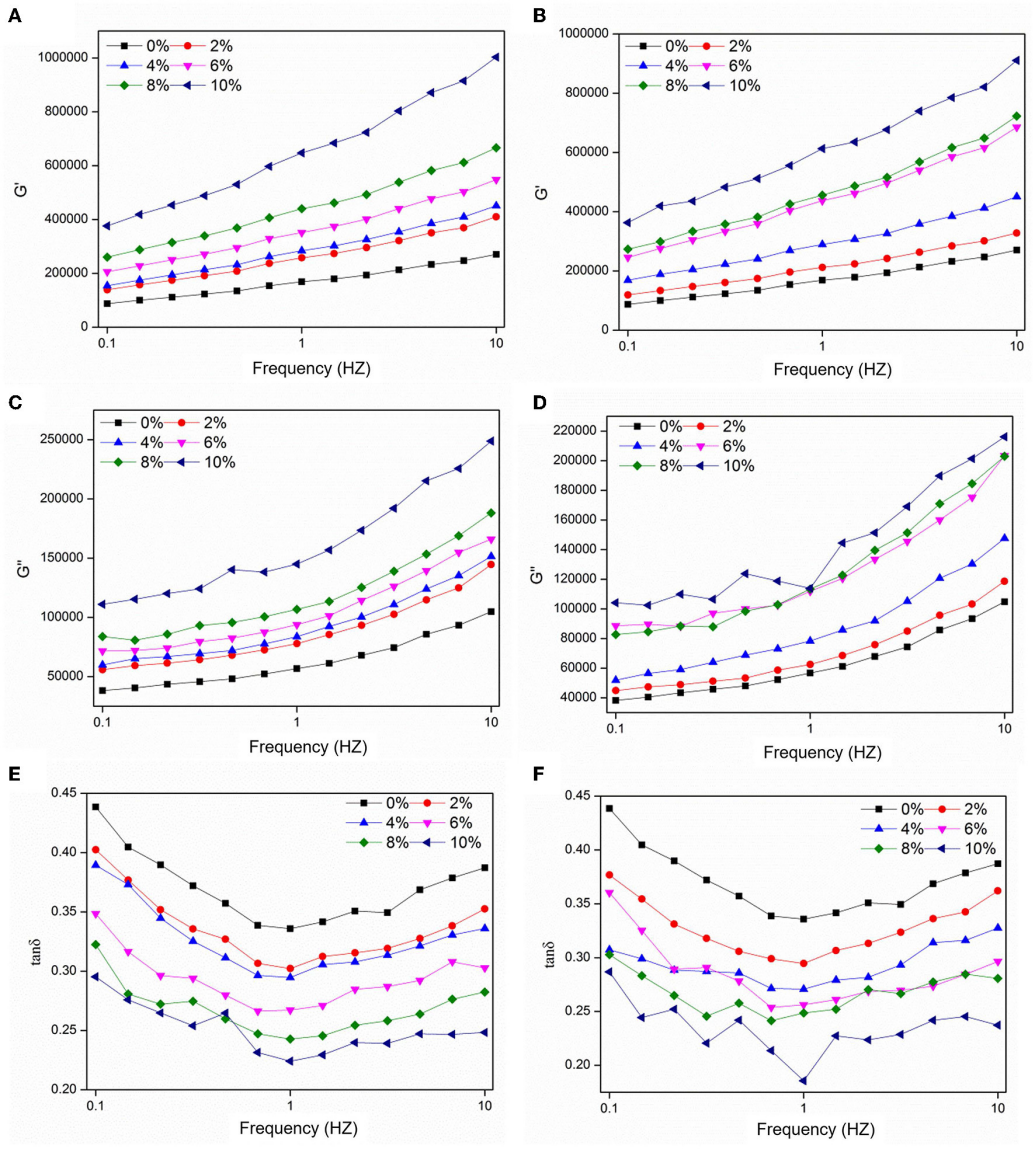
Effect of UWBDF and FWBDF addition on frequency scan curve of dough: **(A,C,E)** dough with UWBDF and **(B,D,F)** dough with FWBDF.

Dough viscoelasticity is related to the network formed by the hydrated gluten, which has an important influence on the processing quality of the dough. As shown in Figure 1, concomitant increases in the UWBDF/FWBDF content led to an overall upward trend in *G*' and *G*” compared with that in the control. However, the same trend of *G*' > *G*” was observed in the presence of different concentrations of UWBDF/FWBDF, indicating that the addition of UWBDF/FWBDF did not change the inherent rheological property of the dough. *G*' reflects the storage modulus and *G*” the loss modulus. This result showed that WBDF could be responsible for the lack of water in the gluten or it acted as a filler in the viscoelastic matrix, thereby increasing the elasticity of the dough (28). Furthermore, the tan δ (*G*”/*G*') showed a pronounced decrease following the inclusion of UWBDF/FWBDF, suggesting an increasing trend in relation to the elasticity. WBDF made the dough a more solid-like material, which might be because of the excessive competition of moisture by WBDF with the existence of a gluten network (29). Similarly, Sui et al. (9) reported that the addition of bran could increase the solid-like behavior of the dough.

However, the *G*', *G*”, and tan δ values of the samples did not change significantly with the same UWBDF/FWBDF. Fermentation promoted the release of some small molecules in WBDF, such as ferulic acid, which were reported to facilitate the formation of larger network structures through oxidative cross-linking with gluten proteins, thereby enhancing the elasticity and stability of gluten networks (9). In this study, the effect of fermentation on the viscoelasticity of dough was not significant, which was probably due to the limited release or the complex interaction of various small-molecule substances with the dough.

#### Creep Sweep

**Figure 2:**
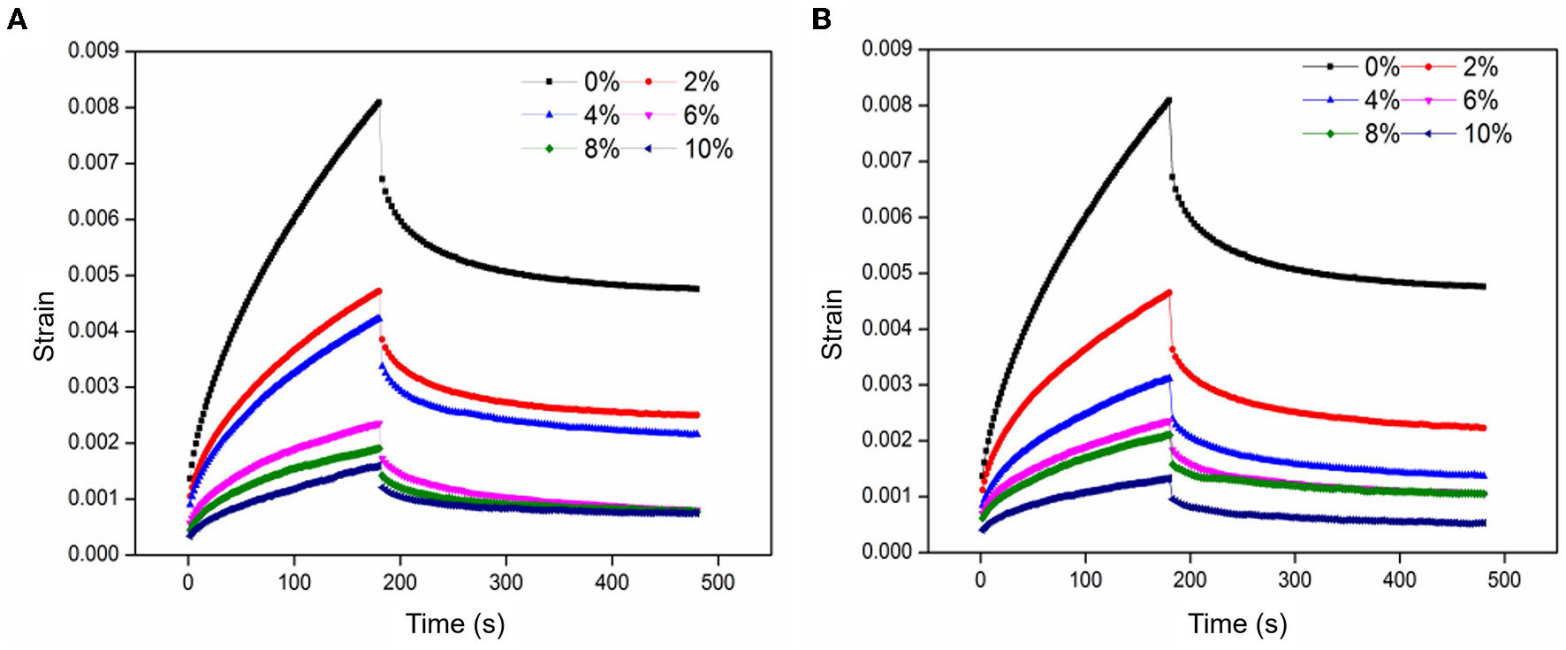
Effect of UWBDF and FWBDF addition on the creep-recovery curve of dough: **(A)** dough with UWBDF and **(B)** dough with FWBDF.

The creep recovery was related to the microstructure changes and the reorientation of chemical bonds, which were usually used to characterize the viscoelastic properties of gluten and dough (30). As shown in Figure 2, the deformation of the sample increased with the extension of time in the creep stage, while the deformation slowly recovered and became stable in the recovery phase after the external force was removed. Increasing UWBDF/FWBDF resulted in a decrease in the creep value, suggesting that dough with WBDF had a firmer texture, and the firmness of the dough positively correlated with the WBDF content. A previous study showed that creep was related to the moisture content of the dough (31). It was inferred that the redistribution of moisture in the dough was responsible for the reduction of the creep value. Furthermore, the recovery rate gradually increased with the addition of WBDF after the external force was removed, indicating that WBDF-containing dough had a more strain-hardening response and stretching stiffness. These results showed that adding WBDF resulted in the dough with higher resistance to deformation and impaired viscoelasticity, thus adversely affecting the quality of the end-use product. No significant change was observed between FWBDF and UWBDF at the same addition level, except for a slight decrease following the addition of 4% FWBDF. Also, the creep of WBDF-containing dough was not significantly affected by fermentation.

### Analysis of Gluten Secondary Structure

The secondary structure of gluten proteins is closely related to gluten network development and the resulting gluten strength. As displayed in Table 1, an overall decrease in the number of α-helices and β-turns and an increase in the number of β-sheets and random coils were observed with the increasing UWBDF/FWBDF ratio. No obvious change was observed in the secondary structure of the gluten following the addition of 2% UWBDF or 2–6% FWBDF. The increased α-helix conformation resulted in a more ordered structure (23, 32). However, a decrease in the α-helix content was accompanied by an increase in the number of random coils; α-helices could be converted into random coils. The dietary fiber competed with the water molecules of the gluten protein, resulting in the redistribution of water in the dough, which caused the breakage of some α-helix hydrogen bonds and the disintegration of the helical structure (23). That is to say, these changes led to the formation of disordered structures. The increased β-sheet and decreased β-turn conformation indicated the formation of a more complex and stronger gluten network. The β-sheet was referred to as the most stable protein conformation (23, 32). This phenomenon could be attributed to the fact that dietary fiber might cause changes in the structure of the gluten proteins (possibility of protein aggregation or abnormal folding), which was more conducive to the formation of a β-sheet structure (33). The result was consistent with the report published by Bock et al. (34), who showed that the addition of wheat bran caused the formation of an intermolecular β-sheet from a β-turn due to moisture redistribution in the dough. However, Zhou et al. (1) demonstrated that the addition of dietary fiber induced the formation of the intermolecular β-sheet from the α-helix than from the β-turn. Also, the changes in structure were related to the interaction among side-chain amino acids or between side-chain amino acid and polysaccharide molecule. The formation of the β-sheet structure in gluten was at least partially derived from the β-turn due to WBDF addition. Within a certain deformation range, the decreased β-turn structure was more unfavorable for the ductility of the peptide chain, resulting in poor gluten extensibility (35). This contributed to the collapse-prone fragile structural properties of the gluten network. Previous studies showed that one of the reasons for the adverse effects of WBDF on the gluten network was the collapse of the β-spiral structure, which was a helical structure composed of repetitive β-turns, into an intermolecular β-sheet (36). This was in good agreement with the findings of this study on dynamic rheological properties.

**Table 1:** Effect of UWBDF/FUWBD addition on the secondary structure of gluten proteins.

**Types**	**Content (%)**	**α-helix (%)**	**β-sheet (%)**	**β-turn (%)**	**Random coil (%)**
UWBDF	0	20.14 ± 1.33^a^	32.34 ± 1.76^de^	36.93 ± 0.93^a^	10.56 ± 1.36^ef^
	2	20.07 ± 1.50^a^	32.68 ± 1.27^de^	36.33 ± 1.42^a^	10.93 ± 0.35^ef^
	4	17.71 ± 1.21^b^	34.27 ± 1.11^cd^	35.81 ± 0.94^a^	12.21 ± 1.26^cde^
	6	16.54 ± 1.21^bc^	36.61 ± 1.21^bc^	32.98 ± 1.86^bc^	13.87 ± 0.55^bc^
	8	14.31 ± 1.33^d^	38.24 ± 1.03^b^	32.22 ± 1.29^cd^	15.23 ± 1.07^ab^
	10	13.14 ± 0.43^d^	40.81 ± 1.08^a^	28.59 ± 1.25^e^	17.46 ± 1.77^a^
FWBDF	0	20.14 ± 1.33^a^	32.34 ± 1.76^de^	36.93 ± 0.93^a^	10.56 ± 1.36^ef^
	2	21.34 ± 1.26^a^	31.27 ± 1.87^e^	37.78 ± 1.23^a^	9.61 ± 1.01^f^
	4	21.11 ± 1.23^a^	31.34 ± 1.07^e^	37.81 ± 1.48^a^	9.74 ± 1.57^f^
	6	20.15 ± 0.57^a^	33.17 ± 1.24^de^	35.47 ± 1.41^ab^	11.21 ± 1.81^def^
	8	17.65 ± 1.78^b^	35.73 ± 1.41^c^	33.16 ± 1.85^bc^	13.46 ± 1.29^bcd^
	10	14.64 ± 1.26^cd^	38.91 ± 1.69^ab^	30.27 ± 1.43^de^	16.18 ± 1.33^a^

*Means in the same column with different small letters indicate a significant difference at P < 0.05*.

Furthermore, with 4–8% addition, the number of α-helices in the FWBDF group was higher than, while the number of β-sheets in the FWBDF group was lower, than that in the UWBDF group. No obvious change was observed in the number of β-turns and random coils between UWBDF and FWBDF, despite a slight drop in the number of random coils following the addition of 4% FWBDF (*P* > 0.05). The effectiveness of fermentation might be associated with the degradation of WBDF. The macromolecular components of WBDF were partially decomposed into small-molecule compounds, thereby changing the structure of WBDF and affecting its physicochemical properties (21). Besides, fermentation softened and hydrolyzed the lignin encapsulated on the cellulose, breaking the multilayered porous network of the plant. FWBDF was found to be easier to grind compared with UWBDF, which might be due to the degradation of the porous structure of WBDF caused by fermentation. Also, all of these changes inevitably affected the secondary structure of gluten and were beneficial in alleviating the destructive effect of dietary fiber on the gluten.

### Water Distribution

**Figure 3:**
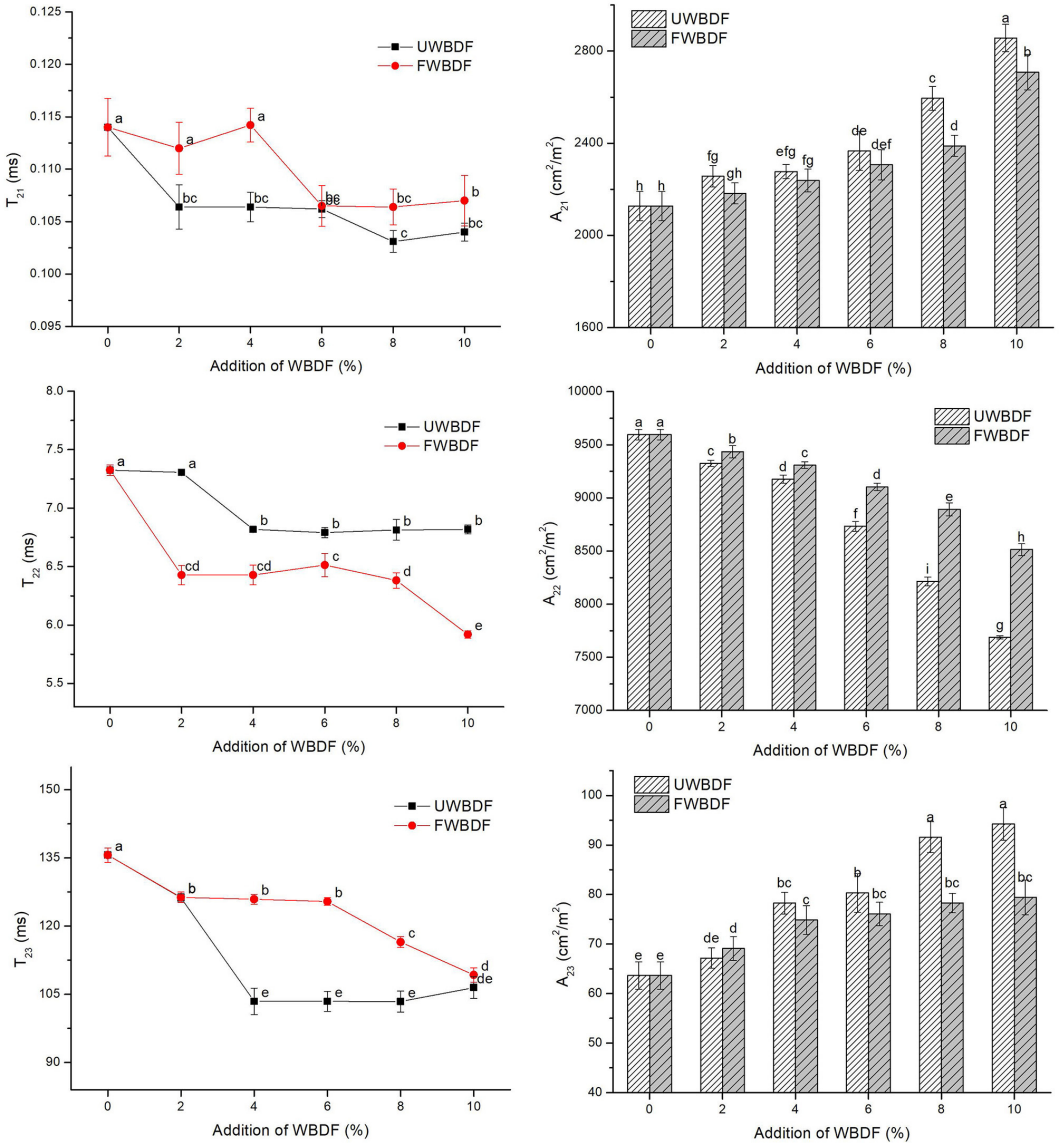
Effect of UWBDF and FWBDF addition on the water distribution of noodles.

Figure 3 shows the influence of UWBDF/FWBDF on the water distributions in noodles. The *T*_21_, *T*_22_, and *T*_23_ represented strongly bound water, weakly bound water, and free water, and their proportions were written as *A*_21_, *A*_22_, and *A*_23_, respectively (37). An overall decrease in *T*_21_, *T*_22_, and *T*_23_ following UWBDF/FWBDF addition indicated that water and macromolecules such as proteins were closely bound. WBDF, containing hydroxyl groups, could bind with water than with protein or starch, thus inhibiting the water flow. The gradual decrease in *A*_22_ and increase in *A*_21_ and *A*_23_ followed the UWBDF/FWBDF addition. The increased *A*_21_ probably contained the water absorbed by WBDF plus intra-granular water in starch; meanwhile, the higher exposure of binding sites in WBDF was also responsible for the increased *A*_23_ in noodles (32). The reduction in *A*_22_ implied that WBDF limited water availability for the interaction between the dietary fiber and the gluten matrix, hindering the formation of the gluten network. Thus, the reduction in *A*_22_ also confirmed the reduced gluten strength in the noodles induced by the addition of WBDF.

The FWBDF group showed an increased *A*_22_ compared with the UWBDF group. As mentioned previously, fermentation was associated with the release of small molecules. It could expose these groups or binding sites so that WBDF contained functional groups such as phenolics, carboxylic acids, and ether linkages, resulting in changes in the water solubility and water-holding capacity of WBDF (38). Meanwhile, the degradation of lignin in WBDF resulted in converting some insoluble dietary fibers into low-soluble soluble dietary fiber and other substances with low polymerization degree and water-binding capacity, further changing the water distribution of the dough. In this way, the ability of dietary fiber to compete with gluten for water was weakened, resulting in a decrease in *A*_22_.

### Cooking, Texture, and Extension Properties of Cooked Noodles

Cooking loss, texture, and extension were important parameters to evaluate the quality of cooked noodles. As shown in Table 2, the addition of UWBDF/FWBDF increased the cooking loss of noodles. The result was in agreement with the findings of Shiau et al. (39). This might be due to the leaching of starch and the dissolution of some proteins due to the loosening of the gluten network. No significant difference was observed in cooking loss at a 2% FWBDF addition level compared with the blank group. Moreover, under the same addition, the cooking loss in the FWBDF group was significantly lower than that in the UWBDF group. Liquor koji and yeast fermentation treatment had positive effects on the cooking and texture properties of noodles, reducing cooking loss and increasing the hardness of whole wheat noodles (40). Cooking loss was the total amount of solid substances left in the cooking process, and the reduction in cooking loss, to some extent, prevented the loss of nutrients. Fermentation improved the cooking quality of WBDF-containing noodles.

**Table 2:** Effect of UWBDF/FUWBD addition on cooking and texture properties of noodles.

**Types**	**Content (%)**	**Cooking property**	**Texture properties**				**Extension properties**	
		**Cooking loss (g/100g)**	**Hardness (g)**	**Cohesiveness**	**Springiness**	**Chewiness**	**Rmax (g)**	**Lmax (mm)**
UWBDF	0	7.72 ± 0.07^f^	4723 ± 74^c^	0.64 ± 0.01^a^	89.19 ± 1.87^a^	2821 ± 26^a^	17.49 ± 0.81^a^	65.52 ± 3.12^a^
	2	8.34 ± 0.30^e^	4280 ± 81^d^	0.58 ± 0.00^bc^	86.68 ± 2.46^ab^	2147 ± 45^d^	16.27 ± 0.53^ab^	56.52 ± 4.24^b^
	4	9.31 ± 0.25^d^	3993 ± 44^e^	0.56 ± 0.01^c^	85.91 ± 0.62^bc^	1956 ± 52^e^	14.19 ± 1.00^c^	47.41 ± 4.53^c^
	6	10.28 ± 0.55^c^	3487 ± 64^f^	0.50 ± 0.01^d^	80.94 ± 1.06d^ef^	1481 ± 92^f^	11.3 ± 0.64^d^	39.61 ± 5.08^de^
	8	10.87 ± 0.09^ab^	3268 ± 81^h^	0.46 ± 0.01^e^	78.36 ± 1.15^f^	1264 ± 11^h^	9.88 ± 0.78^e^	36.61 ± 2.78^e^
	10	11.28 ± 0.28^a^	3157 ± 9^h^	0.44 ± 0.02^e^	79.49 ± 1.15^ef^	1298 ± 68^h^	7.33 ± 0.96^f^	25.83 ± 3.60^f^
FWBDF	0	7.72 ± 0.07^f^	4723 ± 74^c^	0.64 ± 0.01^a^	89.19 ± 1.87^a^	2822 ± 26^a^	17.49 ± 0.81^a^	65.52 ± 3.12^a^
	2	7.34 ± 0.23^f^	5572 ± 23^a^	0.60 ± 0.00^b^	89.07 ± 0.30^a^	2925 ± 69^a^	16.86 ± 0.53^a^	59.00 ± 3.64^ab^
	4	9.05 ± 0.39^d^	5069 ± 10^b^	0.60 ± 0.02^b^	88.47 ± 1.39^ab^	2676 ± 26^b^	15.08 ± 0.56^bc^	51.87 ± 5.42^bc^
	6	9.51 ± 0.20^d^	4638 ± 171^c^	0.58 ± 0.02^bc^	87.50 ± 3.37^ab^	2366 ± 133^c^	12.09 ± 0.72^d^	44.61 ± 4.19^cd^
	8	10.13 ± 0.07^c^	4659 ± 15^c^	0.52 ± 0.01^d^	83.48 ± 1.24^cd^	2018 ± 31^e^	10.92 ± 0.86^de^	39.29 ± 4.69^de^
	10	10.61 ± 0.30^bc^	4639 ± 18^c^	0.52 ± 0.01^d^	82.15 ± 0.84^de^	2022 ± 61^e^	8.53 ± 0.48^f^	28.65 ± 3.26^f^

*Means in the same column with different small letters indicate a significant difference at P < 0.05*.

The texture is one of the key parameters for evaluating the sensitivity of consumers. As shown in Table 2, the increase in the levels of FWBDF added (≤4% addition) improved the hardness of the cooked noodles (*P* < 0.05), while UWBDF addition led to a decrease in hardness. An increase in hardness levels correlated with an increase in dough firmness, most likely due to the formation of the firm protein network analyzed earlier. A previous study showed that fermentation and enzymatic synergy inhibited the damage caused by wheat bran to dough and gluten polymerization (41). The UWBDF addition decreased water availability due to the physicochemical nature of UWBDF to hydrate quickly. Thus, the UWBDF combination with gluten protein resulted in a change in their spatial configuration, preventing them from further cross-linking and resulting in a decrease in the hardness of the noodles. Furthermore, increasing the addition levels of UWBDF/FWBDF resulted in a significant decrease in cohesiveness, springiness, and chewiness, but no significant change was observed in springiness and chewiness with the addition of 2% FWBDF. Rough WBDF was believed to yield an open and disaggregated gluten structure and reduced the cohesiveness, springiness, and chewiness of noodles. These results were similar to the previous findings on noodles by Chen et al. (42). This implied that the redistribution of water and the physical damage due to water-absorbing capacity and hard texture of WBDF might be the main influencing factors (42, 43). These might be the reasons for the changes in the texture of cooked noodles after WBDF addition.

Regarding the extensional properties of cooked noodles, the maximum tensile resistance *R*_max_ and maximum tensile length *L*_max_ decreased remarkably (*P* < 0.05) following the addition of UWBDF/FWBDF. No significant change in the levels was observed after the addition of 2% of FWBDF. No obvious change was observed in the *R*_max_ and *L*_max_ between UWBDF and FWBDF at the same addition level. The decrease in *R*_max_ and *L*_max_ indicated a decrease in the gluten strength and extensibility of noodles, which was basically in agreement with the properties of the dough, implying that noodle quality was related to the gluten conformation. Besides, WBDF with a rougher surface and a higher mechanical strength was responsible for the weakening and disruption of the gluten network by increasing the friction with gluten branches. WBDF created a steric hindrance around the gluten protein, which negatively affected the connection expansion of the gluten network.

In this study, the addition of UWBDF/FWBDF increased the *G'* and *G”* of the dough. Following the analysis of the secondary structure of proteins, the relative abundance of β-sheets and random coils increased. In contrast, the abundance of α-helices and β-turns decreased with the increasing UWBDF/FWBDF ratio. These suggested that the protein conformation changed, resulting in the poor extensibility of gluten and the fragile structural characteristics of the gluten network. Combined with the analysis of noodle texture and extension properties, it was concluded that the change in gluten structure by WBDF was one of the main factors affecting noodle quality. Next, the analysis of water distribution in noodles using low-field nuclear magnetic resonance confirmed that the addition of WBDF inhibited the flow of water in noodles, and the rearrangement of water also affected the quality of noodles. Our analysis showed that FWBDF reduced the destructive effects of dietary fiber on noodle quality by changing the protein conformation and rearrangement of water. Furthermore, the properties of the dough played crucial roles in predicting the quality of noodles.

## Conclusions

This study revealed the effects of FWBDF and UWBDF on the rheological properties of the dough and the quality of noodles. The *G'* and *G”* of the dough increased after adding FWBDF/UWBDF. When the added level of UWBDF reached 6% or that of FWBDF reached 8%, the protein conformation was transformed from α-helix and β-turn to β-sheet and random coil, respectively. Besides, when the addition was in the range of 4–8%, the number of α-helices of FWBDF was higher than that of UWBDF, while the number of β-sheets of FWBDF was lower than that of UWBDF, indicating that adding FWBDF to gluten prevented some of the damage to the gluten network structure compared with UWBDF. The addition of FWBDF/UWBDF facilitated the inhibition of water flow in the noodles; the FWBDF group showed an increased *A*_22_ compared with the UWBDF group. Additionally, an increase in the levels of FWBDF added (≤4% addition) improved the hardness of the cooked noodles, while the addition of UWBDF decreased the hardness of the noodles. Meanwhile, the addition of FWBDF/UWBDF decreased the cooking loss, *R*_max_, and *L*_max_ in the noodles, except for no significant change with the addition of 2% FWBDF. The cooking loss in the FWBDF group was significantly lower than in the UWBDF group. These changes showed that fermentation had positive effects on the cooking and texture properties of WBDF-containing noodles. This study provided new prospects for balancing dietary fiber-rich and quality foods with fermented dietary fiber, thus facilitating a comprehensive understanding of the relationship between WBDF and flour processing. Besides, WBDF could be fermented by various bacterial groups, which was expected to further improve the quality of the end-use products.

## Data Availability Statement

The original contributions presented in the study are included in the article/supplementary material, further inquiries can be directed to the corresponding author/s.

## Author Contributions

LF and AX: conceptualization and writing—original draft. LL: review and editing. JH: conceptualization and supervision. SM: review, editing, and supervision. All authors contributed to the article and approved the submitted version.

## Funding

This work was supported by the Open competition Research Projects of Xuchang University (No. 20220504), Major Science and Technology Projects for Public Welfare of Henan Province (201300110300), Zhongyuan Scholars in Henan (No. 214400510015), and School-land Cooperation Project from Xuchang University (No. 980262).

## Conflict of Interest

The authors declare that the research was conducted in the absence of any commercial or financial relationships that could be construed as a potential conflict of interest.

## Publisher's Note

All claims expressed in this article are solely those of the authors and do not necessarily represent those of their affiliated organizations, or those of the publisher, the editors and the reviewers. Any product that may be evaluated in this article, or claim that may be made by its manufacturer, is not guaranteed or endorsed by the publisher.
